# Stem Cell-Based Therapy and Cell-Free Therapy as an Alternative Approach for Cardiac Regeneration

**DOI:** 10.1155/2023/2729377

**Published:** 2023-11-02

**Authors:** Iwona Deszcz

**Affiliations:** Department of Immunopathology and Molecular Biology, Wroclaw Medical University, Borowska 211, 50-556, Wroclaw, Poland

## Abstract

The World Health Organization reports that cardiovascular diseases (CVDs) represent 32% of all global deaths. The ineffectiveness of conventional therapies in CVDs encourages the development of novel, minimally invasive therapeutic strategies for the healing and regeneration of damaged tissue. The self-renewal capacity, multilineage differentiation, lack of immunogenicity, and immunosuppressive properties of mesenchymal stem cells (MSCs) make them a promising option for CVDs. However, growing evidence suggests that myocardial regeneration occurs through paracrine factors and extracellular vesicle (EV) secretion, rather than through differentiation into cardiomyocytes. Research shows that stem cells secrete or surface-shed into their culture media various cytokines, chemokines, growth factors, anti-inflammatory factors, and EVs, which constitute an MSC-conditioned medium (MSC-CM) or the secretome. The use of MSC-CM enhances cardiac repair through resident heart cell differentiation, proliferation, scar mass reduction, a decrease in infarct wall thickness, and cardiac function improvement comparable to MSCs without their side effects. This review highlights the limitations and benefits of therapies based on stem cells and their secretome as an innovative treatment of CVDs.

## 1. Introduction

According to the World Health Organization, in 2019, 17.9 million people died from cardiovascular diseases (CVDs), predominantly due to heart attack and stroke. The term CVDs mainly refers to coronary artery disease or coronary heart disease, cerebrovascular disease, peripheral artery disease, and aortic atherosclerosis [1]. These diseases are caused by different factors, including aging, stress, diabetes, high blood pressure, atherosclerosis, and viral infections. Lifestyle changes, such as using a low-fat and low-sodium diet, limiting alcohol intake, practicing sports, and quitting smoking are important factors that reduce the risk of CVDs, but are not enough to protect against them. In general, the standard therapy of CVDs is based on pharmacological agents, such as angiotensin-converting enzyme inhibitors, beta-blockers, diuretics, angiotensin receptor blockers, sodium-glucose cotransporter-2 inhibitor, angiotensin receptor neprilysin inhibitor, or aspirin [2]. They are used in case of nonadvance stages of the disease, nevertheless, these agents alleviate symptoms, but do not induce reconstruction of new functioning cardiac tissue. The regenerative potential of the adult heart is insufficient to restore its function after a myocardial infarction (MI), during which millions of cardiomyocytes (CMCs) are irreversibly destroyed. MI causes a noncontractile and nonconductive scar leading to heart failure (HF). Advanced cases of disease require surgical procedures, such as cardiac revascularization therapy with percutaneous coronary intervention and coronary artery bypass grafting, cardiac implantable electronic devices, balloon angioplasty, or a heart transplant [3, 4]. The most advanced therapy is the heart transplant, which is a procedure of last resort for patients with HF who cannot be helped with medical treatment. Unfortunately, a shortage of organ donors and immune matching limits the number of possible heart transplants. Moreover, these patients are forced to take immunosuppressive drugs for the rest of their lives, and, consequently, are at risk of opportunistic infections. This ineffectiveness of conventional therapies in CVDs encourages the development of novel, minimally invasive therapeutic strategies for the healing and regeneration of damaged tissue [5]. Many different innovative approaches have been used in the therapy of CVDs to date, such as cell therapies involving mesenchymal stem cells (MSCs) and their secretomes (the conditioned medium (CM) and extracellular vesicles (EVs)), tissue engineering (3D structures), and gene therapies [6–11]. The group of authors provided worth reading a concise overview of the innovative approaches that are close to or even have reached clinical application for myocardial repair [12]. Moreover, the scientist has been working hard for the past 30 years on xenotranspantations. Interestingly in the USA in January 2022 was conducted first pig-to-human heart transplantation [13]. Notwithstanding the positive outcome of the surgery, the patient died after 2 months, but this success gives hops patients. Over the past decades, multiple experimental studies have been performed investigating the regenerative capability of adult mammalian heart. Since it was highlighted, that during the lifetime human heart is able to replace CMCs (<1% per year), endothelial cells, and mesenchymal cells [14], the stem cell-based therapies, have been tested preclinically and in several early phase trials to either deliver exogenous stem cells or stimulate the endogenous cells to trigger heart regeneration [15–19]. The reason behind the therapeutic viability of MSCs is that they are adult, multipotent stem cells that can be isolated from various tissues, such as bone marrow, adipose tissue, the umbilical cord, dental tissues, placenta, skeletal muscles, and cardiac tissue [20, 21]. Regardless of what tissue the MSCs are isolated from, they must meet the appropriate criteria established by the Mesenchymal and Tissue Stem Cell Committee of the International Society for Cellular Therapy (ISCT) [22]. Cell-based therapies have numerous advantages, but also certain disadvantages. More and more studies prove that stem cells, after transplantation into the heart, regenerate the damaged tissue through paracrine factors and EV secretion, rather than through differentiation into CMCs [23–27]. Growing evidence suggests that stem cell transplantation, despite many disadvantages, is a promising option in CVD therapy. Studies also investigated the use of an MSC-derived secretome in CVDs [27–29]. However, the obtained results were controversial, and opinions differ between researchers. Consequently, it is important to compare the advantages and disadvantages of the two options of CVDs therapy, that is, cell-based therapy and cell-free therapy. This review will focus on the positive and negative effects of therapies involving stem cells and their secretome as an innovative form of CVD treatment.

## 2. Source of Stem Cells

Recent research has focused on stem cell-based therapies using MSCs for cardiac regeneration due to their lack of immunogenicity (lack of MHC class II antigen expression) and their immunosuppressive properties. Bone marrow-derived MSCs (BMSCs) were the first stem cells discovered in 1966 by Friedenstein et al. [30]. They remain the best-studied stem cells to this day, and have been the main source of MSCs for years. However, obtaining cells from the bone marrow involves an invasive and painful procedure. Moreover, the sample contains a very small amount of stem cells. These two reasons have encouraged researchers to look for other sources of stem cells [31]. The ISCT used BMSCs to create the minimum criteria for stem cells [22]. First, stem cells must adhere to plastic in standard culture conditions and express surface molecules, such as CD44, CD73, CD90, and CD105. Second, the cells must be negative for the HLA-DR marker, monocytic markers (CD14 or CD11b, CD79a, or CD19), and hematopoietic lineage markers (CD34 and CD45). Third, MSCs must differentiate into osteoblasts, adipocytes, and chondroblasts in vitro. In 2016, ISCT also established analytic methods to confirm selected MSC markers: quantitative RNA analysis of selected gene products; flow cytometry analysis of functionally relevant surface markers; and a protein-based assay of the secretome [32]. In addition to these three main requirements, MSCs must also promote immunomodulation by trophic factors, such as chemokines, cytokines, growth factors, EVs (e.g., exosomes), glycosaminoglycans, and morphogens, and have an antiapoptotic, antioxidative effect [31, 33, 34]. The immunomodulatory capacity of MSCs also plays an important role in tissue homeostasis and tissue regeneration, but depends on the origin of the stem cells. MSCs are able to regenerate damaged myocardial tissue by stimulating angiogenesis and inducing the neighboring cells to grow or even differentiate. They also regulate the functions of endothelial cells and fibroblasts, which contributes to the reduction of fibrosis that occurs after MI [35]. It is worth mentioning that while stem cells from different sources have many features in common, they also show unique characteristics, which are presented below. This study will focus only on selected MSCs, such as BMSCs, AdMSCs, DSCs, and CSCs, used for regeneration in CVD cases.

### 2.1. Bone Marrow-Derived MSCs

Researchers have made significant progress in the study of BMSCs. BMSCs were shown to exhibit the expression of MSC markers, including CD10, CD13, CD44, CD73, CD90, CD105, CD106, CD146, CD166, STRO-1, and CD49a/CD29, the platelet-derived growth factor receptor (PDGF-R), epidermal growth factor receptor, insulin-like growth factor receptor, and nerve growth factor receptor [20, 21]. Moreover, their high capacity for proliferation and immunomodulation, along with anti-inflammatory and antiapoptotic properties, plays an important role in tissue homeostasis and tissue regeneration through the secretion of a broad spectrum of cytokines and growth factors (interleukin 6 (IL-6), IL-7, IL-8, IL-11, IL-12, IL-14, IL-15, MCP-1, PDGF, vascular endothelial growth factor (VEGF), osteoprotegerin, transforming growth factor beta (TGF-*β*), and TIMP-2) [36]. In addition, BMSCs show a very low immunogenicity due to lack of expression of the major histocompatibility complex MHC-II and a low expression of MHC-I. During in vitro culture, under normal conditions, BMSCs express pluripotent stem cell markers (Oct4, Nanog, Sox2, SSEA, and c-Myc), the chondrogenic marker (type II collagen), osteogenic markers (osteonectin, osteocalcin (OCN), osteopontin (OPN), type I collagen, BMP-2, and BMP-4), glial and neuronal markers (*γ*-enolase, MAP2a,b, *β*III tubulin, and GFAP), and myogenic markers (myogenin, myosin IIa, desmin, actin, and *α*-SMA) [20, 37, 38]. BMSCs are able to self-renew and to differentiate into osteoblasts, adipocytes, chondrocytes, CMCs, hepatocytes, endothelial cells, neural cells, vascular smooth muscle cells, and skeletal muscle cells (SMCs) in vitro [37]. Furthermore, long-term storage and cryopreservation do not affect their morphology, surface marker expression, or importantly, their differentiation and proliferation potential [38]. These features make BMSCs the first and the most promising choice in cardiovascular regenerative medicine.

### 2.2. Adipose Tissue-Derived MSCs

MSCs isolated from adipose tissue express CD10, CD13, CD29, CD44, CD49, CD71, CD73, CD90, CD105, and the STRO-1 protein, and do not express CD11b, CD14, CD19, CD31, CD34, CD45, CD56, CD146, or HLA-DR [39–42]. However, the expression of some markers changes during cultivation. For example, the early passages of adipose tissue-derived MSCs (AdMSCs) are characterized by CD34 expression and low CD105 expression [40, 41]. These are self-renewal-capable cells with a potential for differentiation into adipocytes, osteocytes, chondrocytes, myogenic cells, CMCs, endothelial cells, vascular smooth muscles, hepatocytes, epithelial cells, and neural lineage cells in response to specific culture media [43–45]. AdMSCs secrete cytokines (IL-10, IDO, TNF, IL-6, IL-8, IL-11, IL-12, and IFN-g), growth factors (VEGF, hepatocyte growth factor (HGF), insulin-like growth factor-1 (IGF-1), TGF-*β*, and basic fibroblast growth factor) and prostaglandin E2 [46]. Moreover, they have a strong immunosuppressive effect on NK and B-cells [47]. Unfortunately, AdMSCs have a limited proliferative capacity (four passages); furthermore, their viability and differentiation ability decrease after long-term storage [48]. On the other hand, AdMSCs can be obtained less invasive than BMSCs and with their angiogenic, antiapoptotic potential, secretion of growth factors encourage researchers to use them for cardiac regeneration. Data shows that AdMSCs protect primary CMCs from apoptosis induced by hypoxia, or serum deprivation, furthermore, intramyocardial AdMSC injection improves cardiac function in the healed infarcted heart [49–52]. Moreover, overexpression of HGF and IGF-1 by these cells enhances their regenerative potential by increasing blood flow, decreasing fibrosis, and triggering the cardiac stem cells (CSCs) to migration, proliferation, and differentiation [53, 54].

### 2.3. Dental Stem Cells

Another source of stem cells is the tissues of the oral cavity. A major advantage of this source is easy access to a rich supply of MSCs. Dental stem cells (DSCs) can be divided into dental pulp stem cells (DPSCs), stem cells from human exfoliated deciduous teeth (SHED), periodontal ligament stem cells, dental follicle stem cells, stem cells from the apical papilla, and gingival stem cells [55–60]. As with BMSCs, these cells exhibit a self-renewal capacity and fibroblast morphology. DSCs can differentiate into osteoblasts, odontoblasts, chondrocytes, adipocytes, neurons, endothelial cells, myocytes, CMCs, and hepatocytes in vitro, but their differentiation capacity depends on their place of origin [55, 56, 61–63]. Furthermore, they interact with the immune system cells responsible for adoptive and innate immunity [64]. However, dental pulp tissue contains a higher number of stem cells compared to the other aforementioned dental tissues. DPSCs are relatively easy to isolate, because the teeth are usually obtained via orthodontic procedures as biological waste. DPSCs were first discovered and characterized by Gronthos et al. [55], who isolated them the pulp of impacted third molars. DPSCs meet the requirements of ISCT and, similarly to BMSCs, also exhibit the expression of pluripotent stem cell markers and bone markers [65–67]. Later, Miura et al. [56] identified relatively immature stem cells in the pulp of human exfoliated deciduous teeth with a higher proliferation rate and better osteoinductive ability in vivo than DPSCs.

DPSCs and SHED are frequently used in studies related to regenerative medicine because of their great clinical potential, easy multiplication, and first and foremost, their noninvasive accessibility. Furthermore, they maintain the multipotent properties after both short- and long-term cryopreservation [68]. Data indicate that DPSCs have a stronger immunomodulatory effect and mediate stronger antiapoptotic effects in a hypoxic environment, stimulate angiogenesis, and promote tissue repair much more effectively than MSCs derived from other tissues [38, 39].

### 2.4. Cardiac Stem/Progenitor Cells

**Figure 1:**
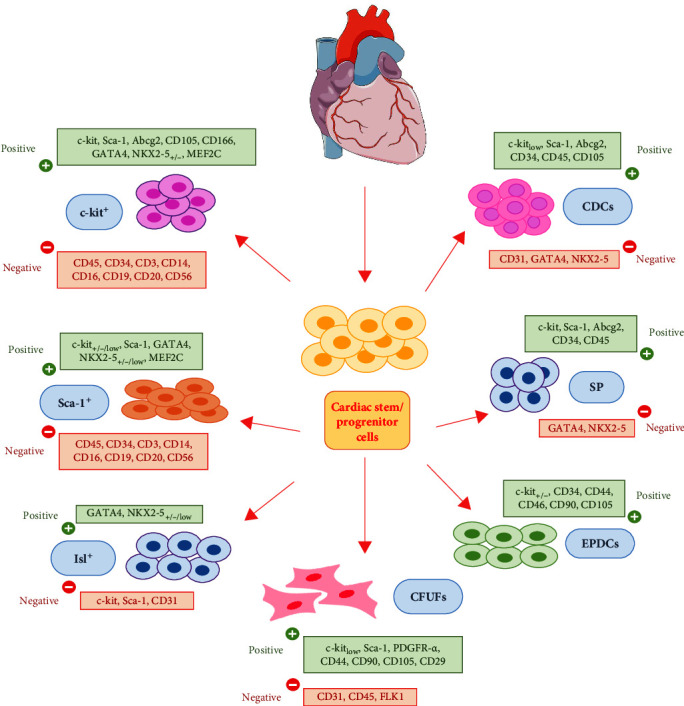
Biomarkers expressed by subpopulations of CSCs resident in the heart. CDCs, cardiosphere-derived cells; SP, cardiac side population cells; EPDCs, epicardium-derived cells; CFUFs, colony forming units fibroblasts.

According to the old paradigm, it was believed, that the heart is a postmitotic organ. However, this thesis has been disproved by researches showing that the adult heart contains a reservoir of small cells expressing stem cell markers such as: c-kit^+^ (type III receptor tyrosine kinase c-kit /CD117), Sca-1^+^ (stem cell antigen 1), and Abcg2 (ATP-binding cassette transporters) [29, 69–71]. These resident tissue-specific endogenous multipotent cells play an important role in heart homeostasis. According to these findings, several populations of CSCs have been identified in the developing and adult heart: CSCs/c-kit^+^ [69, 72]; CSCs/Sca-1^+^ [70, 73]; cardiosphere-derived cells (CDCs) [74]; cardiac side population cells [75, 76]; epicardium-derived cells [77]; PDGFR*α*^+^ expressing CSCs (PDGF-R-alpha)—colony forming units fibroblasts [78], and CSCs/Islet-1^+^ [79]. Presented cells demonstrate a clonogenic, self-renewing, multipotent potential in vitro and in vivo as well as show significant regenerative potential in vivo especially CSCs/c-kit^+^ and CDCs. Moreover, CSCs express Oct3/4, Nanog, Bmi-1, (belonging to stemness markers), and Isl-1, Nkx2.5, MEF2C, GATA4, SOX17, and WT1 (cardiogenic transcriptional factors) [71, 80]. Detailed characteristics of the individual subpopulations of CSCs are presented in Figure 1. In response to tissue damage, hypoxia, ischemia, or another type of cellular stress, in adult heart they are triggered to multiplication and differentiation into new CMCs, endothelial cells, and smooth muscle cells [69, 72, 74]. In in vitro culture CSCs shed into culture medium anti-inflammatory, antiapoptotic (prosurvival) growth factors such as: IGF-1, HGF, TGF-*β*1 superfamily, activins, BMP-10, neuregulin-1, and periostin, which influences cardiovascular regeneration [81]. Data indicate that CSCs/c-kit^+^ have strong immunomodulatory properties and long-term storage, cryopreservation do not affect cells morphology and immunophenotype or, importantly, their differentiation and proliferation potential [69, 74, 81]. Some investigators underline that CSCs are necessary and sufficient to support myocardial cell homeostasis, repair, and regeneration [12, 82, 83]. Unfortunately, obtaining these cells requires an endomyocardial biopsy, which is an invasive procedure associated with a risk of serious complications [84]. Therefore, there is a lot of data showing, that allogeneic CSCs are hypoimmunogenic and have immunomodulatory capacity promoting allostimulation [85, 86]. They promote M2 macrophage polarization, increase regulatory T limphocytes and reduce T cell proliferation [87].

Due to the best regenerative capacity of myocardium the CSCs/c-kit^+^ cells and cardioshpere-derived cells are the most frequently selected CSCs for preclinical researches and clinical trials, therefore in the following chapters they will be taken into account and presented as CSCs.

## 3. Differentiation Potential of MSCs: Stem Cell-Based Therapy

Due to their properties, MSCs have been researched for decades. Knowledge about stem cells is growing, which broadens the perspectives and brings new hopes in regenerative medicine. MSCs from different sources may differentiate under the appropriate conditions into almost any type of cells both in vitro and in vivo. This helps researches to find new strategies and solutions in CVD treatment and to improve the therapeutic efficacy of MSCs. To date, several protocols and their modifications have been established for MSC differentiation into osteoblasts, chondrocytes, and adipocytes in vitro, because differentiated cells must exhibit morphological changes and express the appropriate markers (OCN and OPN; collagen type II; Adipoq, Ppar*γ*, and Fabp4, respectively). The effectiveness of MSC differentiation can be tested by staining the samples with Alizarin Red S for osteogenesis, Oil Red O for adipogenesis, and Alcian Blue for chondrogenesis. Conversely, protocols for MSC differentiation into CMCs are limited, because their transformation into functional CMCs or cardiomyocyte-like cells (CLCs) in in vitro culture is difficult. One reason is that the differentiation potential depends on the place of origin of MSCs [88, 89]. Second, the obtained CMCs/CLCs should have the appropriate mechanical and electrical properties to be engrafted into the injured myocardium. A majority of studies have been performed on BMSCs using various inductor factors. The first successful BMSC differentiation into CMCs was performed in 1999 by Makino et al. [90] using 5-azacytidine (5-aza), the most common cardiac inducer applied recently by researchers. Other authors used a cocktail method including 5-aza, salvianolic acid B (SalB), and a CMC lysis medium to induce BMSCs to acquire the phenotypic characteristics of CMCs [91]. Another study investigated the use of 5-aza and TGF-*β*1 to transform BMSCs and AdMSCs into functional CMCs [92]. The obtained functionality of both MSC types allowed the authors to conclude that AdMSCs induced by TGF-*β*1 were more suitable for CVD stem cell therapy than BMSCs. In turn, Guo et al. [93] briefly presented the regulatory factors that induce BMSCs to differentiate into CMCs. There are also studies showing successful rat/mouse CSC differentiation into CMCs using 5-aza [94, 95]. Interesting results were obtained using extremely low-frequency electromagnetic fields, tuned at the Ca^2+^ ion cyclotron energy resonance (Ca^2+^-ICR), to drive CSCs differentiation toward a cardiac-specific phenotype [96]. Nevertheless, a number of studies have been conducted to investigate CSCs differentiation into spontaneously beating CMCs in vitro [74, 97]. However, most stem cell-based therapies in CVDs are performed using undifferentiated MSCs. Clinical trials demonstrated the safety of autologous BMSC and AdMSC transplantation, as well as improved myocardial function in patients with ischemic heart failure or the ischemic heart disease [98–102]. Furthermore, the TAC-HFT trial revealed that MSCs were more efficient than BM-derived mononuclear cells [103]. The POSEIDON clinical trial showed that an allogenic transplantation of MSCs was effective and as safe as with autologous MSCs, because they did not stimulate any significant donor-specific alloimmune reactions [104]. Unfortunately, there are only a few articles related to DPSC differentiation into CMCs [105, 106] and CVD DPSC-based therapies [23, 107]. Gandia et al. [23] reported that DPSCs transplanted into a rat's heart after MI reduced infarct size, increased capillary density, prevented ventricular remodeling, improved regional contractility, and increased wall thickness. However, scientists are increasingly favoring DPSCs, mostly in neural and oral regeneration, due to their noninvasive accessibility, great clinical potential, and easy multiplication. On the other hand, CSCs seem to be the most suitable cells for myocardial regeneration because, due to their cardiac origin, they can be more easily differentiated into cardiac lineages than other MSCs. Clinical trials using CSCs in patients with left ventricular dysfunction following myocardial infarction (CADUCEUS) also revealed no side effects and showed a reduction of scar size and an increase in myocardium viability [108]. Interesting results were obtained in the CCTRN CONCERT-HF trial, in which patients with HF caused by ischemic cardiomyopathy were treated with autologous BMSCs and CSCs. The trial showed that a combined therapy with both cell types gave the best improvement in the clinical outcome and quality of life, but without scar size reduction or LV function improvement [109]. Moreover, researchers in the clinical trial ALLSTAR obtained encouraging results with allogeneic CSCs therapy in post-MI patients with LV dysfunction. First of all, they showed that intracoronary administration of allogeneic cardioshpere-derived cells is save with no ventricular arrhythmias or myocarditis after 1-month of observation. Second, they revealed that 6 months after CDC infusion had a favorable impact on LV remodeling and a biochemical marker of HF observed [110]. The DYNAMIC trial also showed the safety of intracoronary infusion of allogeneic CSCs in patients with HF and reduced ejection fraction [111]. Despite the promising results of clinical trials performed with MSCs in CVDs, the benefits of stem cells on cardiac function in the clinical setting remain controversial.

## 4. Limitations of Stem Cell-Based Therapy

**Figure 2:**
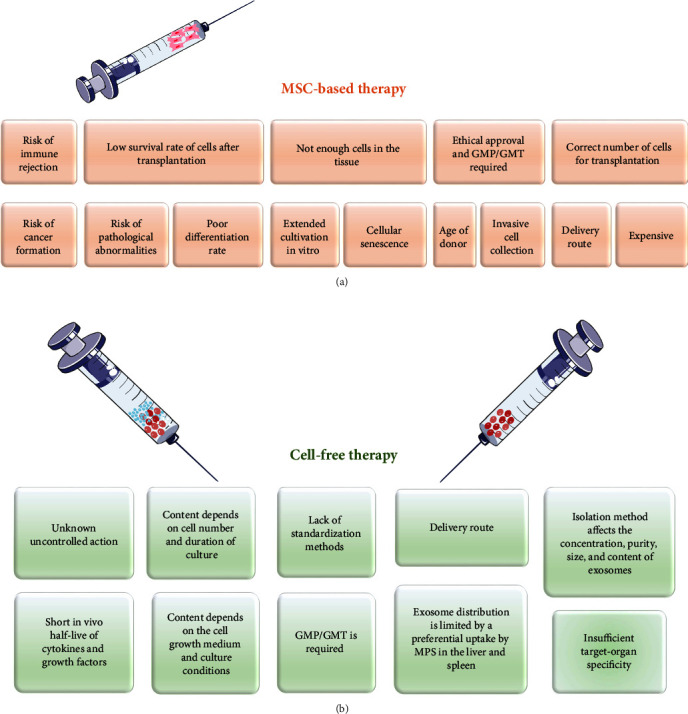
Overview of the disadvantages of (a) MSC-based and (b) MSC-derived secretome therapy.

MSCs are valuable in CVD therapy because of their regenerative potential. Unfortunately, stem cells prove difficult to use due to several limitations, some of which are shown in Figure 2(a). First, every post-MI and HF patient requires a certain number of stem cells. Because the source tissue does not contain enough stem cells for an autotransplant, the cells have to be multiplied in vitro culture. Extended cultivation causes aging of cells, which is presented by the number of cell population doubling (NCPD) and is connected with cellular senescence [112]. It is showed that NCPD goes down with increasing number of passages and it is about 15–30 PD before cells lose the ability to replicate in vitro [72, 113, 114]. Second, long-term growth may affect morphological, phenotypic, and genetic changes, what may influence on the differentiation ability, immunoregulatory potential, heterogeneity, proliferation effectiveness, and survival of MSCs [112, 115]. Aged MSCs change their typical spindle shaped and become enlarged and flattened. In general, every cell division is connected with a shortening of chromosomal telomeres, until cell achieves about 50 divisions and after this stops and becomes senescent (known as the Hayflick and Moorhead [116] limit, discovered by Hayflick in human fibroblasts derived from fetuses). When the cell enters the G0 phase, is irreversibly arrested—is not able to divide, but remains metabolically active. Third, the senescent cell can influence surrounding cells, by secreting different senescence-associated secretory phenotypes (SASPs) factors, what may affect tissue regeneration by spreading senescence. SASPs, consist of inflammatory cytokines, chemokines, growth factors, matrix remodeling proteases, and lipids [117, 118]. In addition, the early senescence may cause changes in the expression of proinflammatory factors and a reduced immunomodulatory potential. Some studies showed that during prolonged cultivation (passage 15), AdMSCs lost their proliferation capacity, and their morphology changed, but they did not cause immunophenotype changes [119–121]. Interestingly, AdMSCs were shown to not lose their differentiation potential into the bone, cartilage, and fat at passage 15, but the expression levels of stemness-related genes, Sox2, Oct4, Nanog, and c-myc decreased [119–121]. A study performed with DPSCs showed that proliferation slowed down after passage 13 and stopped after passage 17 [122]. Moreover, prolonged DPSC culture downregulated the Sox2, Oct4, and Nanog transcription factors, which are responsible for the stem cell phenotype. Interestingly, Vicinanza et al. [82] showed that CSCs unlike other MSCs have proliferative activity above 65 passages and maintained a stable phenotype without growth arrest, senescence, or downregulation of stemness and cardiac gene expression. However, they also pointed out that freshly isolated CSCs (passage 1) present a low proliferation rate [123]. Scalise et al. [124] confirmed that CSCs have high clonogenic potential in vitro with more than 50 passages without cellular and/or molecular senescence.

CVDs are often referred to as *diseases of affluence*, and their prevalence increases with age. There are reports on the relationship between donor age and stem cells proliferation, differentiation potential, and amount in the source tissue decline and consequently affects the outcome of stem cell-based therapy. Regrettably, aging is also connected with increasing cellular senescence what impedes tissue regeneration. Moreover, CVDs are primarily associated with tissue hypoxia, which leads to their destruction by cell death. The stress stimulus may negatively affect cell therapy, and with older patients, may only provide a minor outcome. BMSCs from older donors (<40 years old) react worse to such difficult conditions, with poorer survival and angiogenic growth factor expression, than those from younger donors [125]. In contrast to stem cells collected from younger donors, they proliferate slower and become senescent after four or five passages [126, 127]. Research indicates that aging affects the differentiation potential of BMSCs into osteoblasts, but does not significantly change their myogenic differentiation capability [128]. There are conflicting reports about the correlation between donor age and AdMSC regenerative potential. Some studies showed that aged AdMSCs had reduced viability, proliferation, and differentiation potential compared to young MSCs [129–131]. Other studies found no relationship between donor age and cell features [132, 133]. There are also reports showing age-related changes in the features of DPSCs. The proliferation capacity, self-renewal, and differentiation potential of DPSCs and stem cell marker expression decrease with donor age, but with no changes in cell morphology [134, 135]. Most studies show that MSCs collected from older donors (>55 years old) differentiated better into adipose tissue cells than those collected from younger patients (<40 years old). Regrettably, endogenous myocardial survival and repair is decreasing with biological age, which is associated, primary, with CSCs aging and cellular senescence. According to findings, half of CSCs isolated from older patients (>70 years old) with CVD turned out to be senescent, without the capability to proliferate, differentiate, and especially without regenerative ability of cardiac tissue [136–138]. Furthermore, Sharm et al. [139] indicated that expression levels of stemness-related genes, Sox2, Oct3/4, and Nanog decreased in adult CSCs compared to neonatal CSCs (<1 month). They showed also that CSCs derived from older donors have lower secretion of angiogenic factors and in vivo regeneration ability. However, not only age may affect the biological potential of donor MSCs, but similarly medications (i.e., anticancer drugs and their cardiotoxicity), and other morbidities like autoimmunological diseases (such as diabetes mellitus, rheumatoid arthritis, or systemic lupus erythematosus) [140–142]. Several reports have demonstrated, that diabetes impairs MSCs proliferation, differentiation, and angiogenic potential [140, 143, 144]. Moreover, diabetes may accelerate the formation and accumulation of senescent cells leading to tissue dysfunctions and consequently to CVDs [145]. In general, these features, together with morbidities *milieu*, which damage the population of endogenous CSCs, affect the regenerative capacity of stem cells in damaged myocardium.

A hypothesis has been put forward that in the hypoxic tissue, some of the transplanted cells migrate to the lungs while the remainder die through apoptosis [24–26]. Consequently, research indicates that MSCs regenerate the injured myocardium through a paracrine effect, rather than CMC differentiation. This theory may be confirmed by the next limitation—the route of cell delivery, which also affects the success rate of the stem cell-based therapy of CVDs. Two main routes can be distinguished: intravenous and local (intracoronary and intramyocardial) transplantation [146]. Their limitation has been shown in studies that proved that stem cells after injection are entrapped in the liver, lungs, and spleen or migrate there after injection, regardless of whether they were given intravenous or intramyocardial [147–149]. Even in the punctures after injection may transient inflammation occur, which the long-term presence may give origin of fibrosis in injection site. In addition, over dose of cells can cause coronary artery occlusion, myocardial edema resulting in regional MI [150]. It needs to be kept in mind, that every open-chest surgery is usually associated with a high risk of perforation in the setting of acute ischemia, necrosis, as well as secondary injuries and infections, therefore it is better to avoid them [146]. Moreover, there are studies demonstrating the possibility of pathological abnormalities after BMSC injections, including the calcification/ossification of infarcted myocardium [151]. This finding indicates that MSCs may undergo undesired differentiation after transplantation to the injured tissue. Furthermore, the occurrence of cardiac arrhythmia may be caused by skeletal myoblast, as well as, MSCs (but to a lesser extent) used for myocardial regeneration [152, 153]. The studies showed that host CMCs have different electrophysiology than SMCs, which compromises heart bioelectrical heterogeneity [154]. Transplanted stem cells electrochemical coupling must be homogenous with host CMCs. However, there is growing evidence showing no side effects related to arrhythmia after MSCs therapy [155, 156]. A promising candidate for cardiac regenerative cell-based therapies are iPSCs (induced pluripotent stem cell), but SMCs may cause arrhythmia and they carry a risk of teratoma [157, 158].

In addition to the biological features of MSCs, all procedures connected with the collection and manipulation of MSCs must be ethically approved and follow the Good Manufacturing Practice (GMP) in the European Union No. 1394/2007 and the Current Good Tissue Practice requirements (GTP) in the United States Code of Federal Regulation (CFR) Title 21 CFR 1271, as they are considered drugs [21, 159–161]. These procedures are long and expensive, because every step, from collection from the donor, through transport and storage of samples, to administration to the patient, undergoes quality control. Special laboratories are needed with appropriate equipment, reagents, and supplies meeting the requirements of the GMP/GTP (Figure 1(a)). In general, laboratories should have an air purity class of A–D, with appropriate temperature, humidity, and pressure to prevent particles and microorganisms from multiplying. The staff should be qualified and trained according to the GMP/GTP. They must also be careful to prevent crosscontamination between samples; consequently, high-efficiency particulate absorbing filters should be used, and the laboratory and equipment should be thoroughly sterilized. As has already been mentioned, every cell collection protocol must pass quality control. In addition, BMSC, ADSC, and CSC collection is invasive and expensive. Due to the above limitations of MSCs, researches have begun looking for alternatives in CVDs therapy.

## 5. Mesenchymal Stem Cell-Free Therapy—MSCs Secretome

Cell-free therapies are gaining popularity in regenerative medicine. Growing evidence indicates that MSCs promote myocardial healing through paracrine secretion, rather than stem cell differentiation after transplantation [27, 162]. Research shows that stem cells, depending on their source, secret or surface-shed into their culture media various cytokines, chemokines, growth factors, and anti-inflammatory factors, including IL-1, IL-6, VEGF, the HGF, IGF-1, IGF-2, SDF-1, and TGF-1 [34, 163]. These paracrine factors constitute an MSC-conditioned medium (MSC-CM) or the secretome [164]. The MSC secretome affects cell viability, proliferation, apoptosis, angiogenesis, fibrosis, and immune responses. MSCs in different environments also secrete EVs into the medium, which are membrane-bound vesicles that can be divided based on their origin into exosomes (EXs), microvesicles (MVs), and apoptotic bodies [165, 166]. Apoptotic bodies are the largest among EVs, ranging from 0.5 to 2 *µ*m. Because they develop from the last phase of apoptotic cell death, they may contain a wide variety of cellular components, such as micronuclei, chromatin remnants, cytosol portions, degraded proteins, DNA fragments, or even intact organelles [166]. MVs emerge through membrane budding, and compared to EXs, are heterogeneous in size and measure between 100 and 500 nm. They contain phosphatidylserine, metalloproteinases, some integrins, and selectins (P-selectin) [167]. Conversely, EXs derived from multivesicular bodies are the smallest, measuring 40–120 nm [167]. Their molecular content depends on the cell type, and mostly comprises lipids, proteins (e.g., heat shock proteins such as HSP60, HSP70, and HSP90), metabolites, DNA, mRNAs, microRNAs (miRNAs), and long noncoding RNAs [21, 162, 165]. EXs express tetraspanins (CD9, CD63, CD81, and CD86), which are involved in the fusion between the EXs and the recipient cells [162]. Depending on the content, EXs may affect the physiological and pathological functions of the recipient cells (immune responses and survival mechanisms). They are also responsible for intracellular communication.

**Figure 3:**
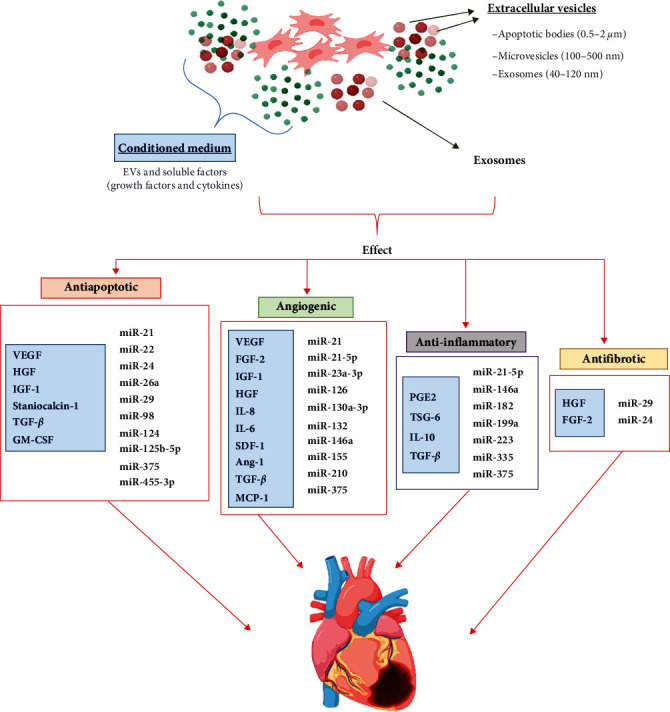
The most common factors of MSC-derived secretomes are associated with antiapoptotic, anti-inflammatory, antifibrotic, and angiogenic/neovascularization processes in heart regeneration. The use of a conditioned medium and exosomes enhances cardiac repair through resident heart cell differentiation, proliferation, scar mass reduction, a decrease in infarct wall thickness, and cardiac function improvement.

As shown in Figure 3, MSCs secret bioactive molecules, which are able to improve tissue repair. In CVDs, the secretome/CM is involved in myocardial protection, cardiac remodeling, neovascularization, and stimulation of CSC differentiation [168]. The therapeutic effect of the MSC-CM was confirmed in models of ischemic cardiac injury and, most importantly, was proven to be comparable to stem cell-based therapy [169–171]. These findings may help to eliminate costly procedures involving stem cell isolation and culture. This means that the MSC-CM may become an off-the-shelf material for immediate application that minimizes surgical invasiveness. Moreover, the CM is easy to produce, because no special culture media or equipment are needed. Unlike stem cells, the secretome is easier to store, because it does not need cryopreservation with special reagents; rather, it is sufficient to freeze or lyophilize it, which makes transport less complicated. It is worth noting that, in addition to the positive effects of the secretome (CM and EXs) on the postinfarcted heart, xenogeneic and allogenic exosome treatment showed no immunological response [172, 173]. This opens up new perspectives for CVD treatment without donor-recipient matches.

Timmers et al. [174, 175] were the first to demonstrate the cardioprotective effects of the BMSC-CM in a pig model of myocardial ischemia and reperfusion injury. They observed a reduction of apoptosis and myocardial infarct size and an increase in capillary density in the border areas, which was confirmed by preserved LV dimensions and cardiac function [174, 175]. Findings about the antiapoptotic effect of the AdMSC-CM on CMCs under hypoxic conditions vary. Lee et al. [176] indicate that the AdMSC-CM suppresses hypoxia-induced CMC apoptosis exhibited through an increase in the level of the p53 upregulated modulator of apoptosis (PUMA) and p-p53 expression and a decrease in the level of the B-cell lymphoma 2 protein and the expression of the fibrosis-related proteins ETS-1, fibronectin, and collagen type 3. The same authors also showed that AdMSC-CM administration reduced cardiac apoptosis and fibrosis in an I/R injury through the miR-221/222/PUMA/ETS-1 pathway and p38 MAPK/NF*κ*B signaling pathway in vitro and in vivo. Other studies showed that the AdMSC-CM from hypoxia-induced AdMSCs contained higher amounts of VEGF, HGF, FGF-2, TGF- *β*, IL-1, and stromal-derived factor-1 (SDF-1 or CXCL12) compared to normoxia [171, 177]. Furthermore, hypoxic AdMSC-CM improved MI cardiac tissue damaged in an in vivo model through the ameliorate apoptosis of CMCs accompanied by changes in JNK signal activation [171, 177]. On the other hand, Yee-Goh et al. [27] observed in an in vitro study that the AdMSC-CM did not reduce apoptosis in hypoxia-induced CMCs, in contrast to CPC-CM, which increased the level of the HIF-1*α* protein in CMCs. In addition, both cell types secreted factors that affected angiogenesis. The authors also suggested that not all MSC-CMs are able to inhibit hypoxia-induced CMC apoptosis [27]. An in vitro study showed that the neonatal CSC-CM stimulated angiogenesis, decreased oxidative stress-induced apoptosis, and promoted CMC proliferation [139]. Subsequently, in an in vivo model, the authors observed post-MI myocardium recovery using the CSC-CM in a rat model manifested through the stimulation of CMC proliferation, reduction of fibrosis and inflammation, and improvement of neovessel density. It is worth pointing out that only Yamaguchi et al. [178] investigated the effect of the DSC-CM on cardiac tissue. They showed that the DSC-CM protected the mouse heart from hypoxic injury by suppressing inflammatory cytokines, such as TNF-*α*, IL-6, and IL-*β*, as confirmed by improved cardiac function after I/R and attenuated MI. They also showed that the antiapoptotic action of the SHED-CM was stronger than that of the BMSC-CM and AdMSC-CM in CMCs, which may have been caused by higher HGF and VEGF concentrations. To date, no more original research investigating the regenerative effect of DSCs-CM on cardiac tissue has been conducted.

Recently, researchers have indicated EXs isolated from the MSC-CM as potent paracrine vectors for the regeneration of post-MI myocardium with recovery of heart function through an antiapoptotic, antifibrotic, anti-inflammatory, and proangiogenic effect, and resident heart cell differentiation (Figure 2). A majority of studies investigating the effect of MSC-EXs (BMSCs, AdMSCs, and CSCs) in vivo models of ischemic cardiac injury confirmed a reduction of infarct size, inflammation, and CMC hypertrophy, and inhibition of cell apoptosis and tissue fibrosis, which contributed to cardiac function improvement (increased LVEF) [172, 179–184]. The injected EXs were able to reach both the neighboring cells and the cells in distant districts, because their lipid bilayer protected their cargo against degradation in the extracellular space. Researchers began to investigate the molecular mechanisms responsible for MSC-EX-mediated post-MI regeneration. It was found that EXs contained one of the most important molecular factors controlling cardiac repair, miRNA [185]. Zhao et al. [179] demonstrated that the miR-182 contained in BMSC-EXs reduced inflammation in infarcted myocardium, as confirmed by changes in the concentration of pro- and anti-inflammatory cytokines (a decrease in IL-6 and an increase in IL-10), as well as a reduced presence of neutrophils. The authors established that miR-182 mediated macrophage polarization through the TLR4/NF-*κ*B/PI3K/Akt signaling cascades. Other authors demonstrated that the BMSC-EX is rich in miR-210, which is responsible for angiogenesis in the ischemic heart [186]. In turn, miR-29 and miR-24 may prevent tissue fibrosis, inhibit CMC apoptosis, attenuate infarct size, and reduce cardiac dysfunction [180]. Likewise, it was found that the therapeutic benefit of the cardiosphere-derived cell exosome may be due to its rich content of miR-24 and miR-146a [182]. In turn, the antiapoptotic and proangiogenic effect of miR-210 and miR-132, contained in the CSC-EX, improved cardiac function after MI [187]. However, despite the proven regenerative potential of EXs for infarcted myocardium, some researchers suggest that MSC secretomes should be considered jointly for therapeutic purposes, rather than individually, that is, divided into the CM and EXs [139, 187]. These authors emphasized that EVs are an active component of the paracrine secretion of stem cells, and that the MSCs-derived secretome is essential for myocardial functional recovery. A strategy involving miR used in CVD therapy is shown in Figure 3.

## 6. Limitation of the MSC Secretome Therapy

Despite the benefits of the MSC secretome in CVD therapy, we are still far from reaching a point where it can be used to treat patients. At least some of the challenges presented in Figure 2(b) must first be eliminated before the MSC-CM can be approved for use in regenerative medicine. The most pressing issue is the lack of standardization methods for the acquisition of the MSC-CM and MSCs-EXs and their quality control—there are no GMP methods. The content of the CM depends on the number of cells or even the duration of culture. The secretory potential of MSCs was shown to depend on the cell growth medium, its content, and primarily, culture conditions [163]. Numerous studies show that hypoxia affects the paracrine effect of MSCs by changing the level of cytokines and growth factors shed into the medium. Moreover, the method of isolation affects the concentration, purity, size, and content of EXs [163, 188, 189]. Consequently, it is important to standardize the manufacturing protocols for obtaining CM and high-purity EX samples with the characterized features. In addition, confirmed findings about the content of the MSC-CM and MSC-EXs should be used to aid the selection of treatment procedures for individual diseases. The preceding section of this article mentioned that the secretome is easier to store than the cells. However, there are reports that demonstrate the challenges of EV freezing [190–192]. Second, the CM contains cytokines and growth factors, which in general are unstable molecules with short in vivo half-lives, which enforces more frequent dosing [193]. Conversely, the distribution of EXs into the heart is limited by a preferential uptake on the part of the mononuclear phagocyte system in the liver and spleen [194]. Moreover, the secretome is associated with insufficient target-organ specificity. Consequently, various modifications of EXs are carried out, such as engineering EXs with targeting proteins on the membrane, which enables them to reach the target cells (CMCs) [195, 196]. An appropriate delivery and controlled, sustained release of secretome factors may be aided with different scaffolds, such as hydrogels [197]. Last but not least, a major challenge in cell-free therapy is the lack of knowledge about uncontrolled CM and EX action. Their positive effect on the target tissue is known, but the long-term consequences of their use on the human body still need to be investigated. Unfortunately, a study has confirmed the potential of the MSC-CM to induce tumor growth comparable to stem cells [198]; consequently, further research is needed.

## 7. Conclusions

Heart diseases are a problem in developing countries. For decades, researchers around the globe have been looking for the best solution for heart regeneration. A promising option seemed to be therapy based on stem cells due to their regenerative, neovascularization, and immunoregulatory capacity. Unfortunately, MSC-based therapies involve time-consuming proliferation, as well as obtaining permissions from bioethics committees and legal bodies. In addition, a majority of heart diseases are associated with senior patients, and age has been shown to affect the patient's stem cells. While this issue can be solved with an allograft, donor matching is needed, which comes with a risk of immune rejection. Furthermore, because the postinfarction cardiac milieu is unfavorable, most cells disappear after MSC transplantation. Many researchers have attempted to find a solution to this issue. Several preclinical studies have demonstrated myocardial regeneration using an MSC-derived secretome transplantation comparable with MSC-based therapy, but without its side effects. It is worth noting that the crucial advantage of the MSC-derived secretome is the lack of immune response of the recipient after the injection. Their nonimmunogenic properties allow for the use of allo- or even xenografts, making them more attractive than stem cells.

This review presented the benefits and limitations of MSCs and MSC-derived secretome transplantations in order to answer the question: Will stem cell-based therapy or cell-free therapy replace conventional treatment for heart diseases? The question is not easy to answer. Both types of therapy seem to be promising options, but too little time has passed to determine their long-term outcomes. Nonetheless, in the future, physicians may need to decide which mode of treatment to use. The decision should be adapted to each patient, including his or her general health and comorbidities, because these factors determine the success of both conventional therapy and new procedures. Stem cells and their secretome open up new possibilities for heart treatment. However, extensive research still needs to be performed before stem cell-based or cell-free therapy can support the conventional treatment of CVDs, let alone replace it.

## Data Availability

No data is available for this study.
